# Supplementary material for: OpenMM 7: Rapid development of high performance algorithms for molecular dynamics
Source: PLoS Comput Biol. 2017 Jul 26;13(7):e1005659. doi: 10.1371/journal.pcbi.1005659 (PMC5549999; doi:10.1371/journal.pcbi.1005659)
Supplement: S1 Source Code — (ZIP) [file pcbi.1005659.s002.zip › OpenMM-7.0.1-Source/docs/api-c++/generated/OpenMM.RPMDIntegrator.html]

RPMDIntegrator — OpenMM @OPENMM\_MAJOR\_VERSION@.@OPENMM\_MINOR\_VERSION@ documentation


# RPMDIntegrator¶

*class* `OpenMM::``RPMDIntegrator`¶
:   This is an `Integrator` which simulates a `System` using ring polymer molecular dynamics (RPMD). It simulates many copies of the `System`, with successive copies connected by harmonic springs to form a ring. This allows certain quantum mechanical effects to be efficiently simulated.

    By default this `Integrator` applies a PILE thermostat to the system to simulate constant temperature dynamics. You can disable the thermostat by calling setApplyThermostat(false).

    Because this `Integrator` simulates many copies of the `System` at once, it must be used differently from other Integrators. Instead of setting positions and velocities by calling methods of the `Context`, you should use the corresponding methods of the `Integrator` to set them for specific copies of the `System`. Similarly, you should retrieve state information for particular copies by calling `getState()` on the `Integrator`. Do not query the `Context` for state information.

    You can optionally specify a set of “ring polymer contractions”, by which different force groups are evaluated on different numbers of copies, instead of computing every force on every copy. This can be much more efficient, since different forces may vary widely in how many times they must be evaluated to produce sufficient accuracy. For example, you might simulate a 32 copy ring polymer and evaluate bonded forces on every copy, but contract it down to only 6 copies for computing nonbonded interactions, and down to only a single copy (the centroid) for computing the reciprocal space part of PME.

    Methods

    |  |  |
    | --- | --- |
    | `RPMDIntegrator` | Create a `RPMDIntegrator`. |
    | `RPMDIntegrator` | Create a `RPMDIntegrator`. |
    | `getNumCopies` | Get the number of copies of the system being simulated. |
    | `getTemperature` | Get the temperature of the heat bath (in Kelvin). |
    | `setTemperature` | Set the temperature of the heat bath (in Kelvin). |
    | `getFriction` | Get the friction coefficient which determines how strongly the system is coupled to the heat bath (in inverse ps). |
    | `setFriction` | Set the friction coefficient which determines how strongly the system is coupled to the heat bath (in inverse ps). |
    | `getApplyThermostat` | Get whether a thermostat is applied to the system. |
    | `setApplyThermostat` | Set whether a thermostat is applied to the system. |
    | `getRandomNumberSeed` | Get the random number seed. |
    | `setRandomNumberSeed` | Set the random number seed. |
    | `getContractions` | Get the ring polymer contractions to use for evaluating different force groups. |
    | `setPositions` | Set the positions of all particles in one copy of the system. |
    | `setVelocities` | Get the velocities of all particles in one copy of the system. |
    | `getState` | Get a `State` object recording the current state information about one copy of the system. |
    | `getTotalEnergy` | Get the total energy of the ring polymer. |
    | `step` | Advance a simulation through time by taking a series of time steps. |

    `RPMDIntegrator`(int *numCopies*, double *temperature*, double *frictionCoeff*, double *stepSize*)¶
    :   Create a `RPMDIntegrator`.

        |  |  |
        | --- | --- |
        | Parameters: |  |

        - **numCopies** – the number of copies of the system that should be simulated
        - **temperature** – the temperature of the heat bath (in Kelvin)
        - **frictionCoeff** – the friction coefficient which couples the system to the heat bath (in inverse picoseconds)
        - **stepSize** – the step size with which to integrator the system (in picoseconds)

    `RPMDIntegrator`(int *numCopies*, double *temperature*, double *frictionCoeff*, double *stepSize*, *const* std::map<int, int> &*contractions*)¶
    :   Create a `RPMDIntegrator`.

        |  |  |
        | --- | --- |
        | Parameters: |  |

        - **numCopies** – the number of copies of the system that should be simulated
        - **temperature** – the temperature of the heat bath (in Kelvin)
        - **frictionCoeff** – the friction coefficient which couples the system to the heat bath (in inverse picoseconds)
        - **stepSize** – the step size with which to integrator the system (in picoseconds)
        - **contractions** – the ring polymer contractions to use for evaluating different force groups. Each key in the map is the index of a force group, and the corresponding value is the number of copies to evaluate that force group on. If no entry is provided for a force group (the default), it is evaluated independently on every copy.

    int `getNumCopies`() *const*¶
    :   Get the number of copies of the system being simulated.

    double `getTemperature`() *const*¶
    :   Get the temperature of the heat bath (in Kelvin).

        |  |  |
        | --- | --- |
        | Returns: | the temperature of the heat bath, measured in Kelvin |

    void `setTemperature`(double *temp*)¶
    :   Set the temperature of the heat bath (in Kelvin).

        |  |  |
        | --- | --- |
        | Parameters: |  |

        - **temp** – the temperature of the heat bath, measured in Kelvin

    double `getFriction`() *const*¶
    :   Get the friction coefficient which determines how strongly the system is coupled to the heat bath (in inverse ps).

        |  |  |
        | --- | --- |
        | Returns: | the friction coefficient, measured in 1/ps |

    void `setFriction`(double *coeff*)¶
    :   Set the friction coefficient which determines how strongly the system is coupled to the heat bath (in inverse ps).

        |  |  |
        | --- | --- |
        | Parameters: |  |

        - **coeff** – the friction coefficient, measured in 1/ps

    bool `getApplyThermostat`() *const*¶
    :   Get whether a thermostat is applied to the system.

    void `setApplyThermostat`(bool *apply*)¶
    :   Set whether a thermostat is applied to the system.

    int `getRandomNumberSeed`() *const*¶
    :   Get the random number seed. See `setRandomNumberSeed()` for details.

    void `setRandomNumberSeed`(int *seed*)¶
    :   Set the random number seed. The precise meaning of this parameter is undefined, and is left up to each `Platform` to interpret in an appropriate way. It is guaranteed that if two simulations are run with different random number seeds, the sequence of random forces will be different. On the other hand, no guarantees are made about the behavior of simulations that use the same seed. In particular, Platforms are permitted to use non-deterministic algorithms which produce different results on successive runs, even if those runs were initialized identically.

        If seed is set to 0 (which is the default value assigned), a unique seed is chosen when a `Context` is created from this `Force`. This is done to ensure that each `Context` receives unique random seeds without you needing to set them explicitly.

    *const* std::map<int, int> &`getContractions`() *const*¶
    :   Get the ring polymer contractions to use for evaluating different force groups. Each key in the map is the index of a force group, and the corresponding value is the number of copies to evaluate that force group on. If no entry is provided for a force group, it is evaluated independently on every copy.

    void `setPositions`(int *copy*, *const* std::vector<Vec3> &*positions*)¶
    :   Set the positions of all particles in one copy of the system.

        |  |  |
        | --- | --- |
        | Parameters: |  |

        - **copy** – the index of the copy for which to set positions
        - **positions** – the positions of all particles in the system

    void `setVelocities`(int *copy*, *const* std::vector<Vec3> &*velocities*)¶
    :   Get the velocities of all particles in one copy of the system.

        |  |  |
        | --- | --- |
        | Parameters: |  |

        - **copy** – the index of the copy for which to set velocities
        - **velocities** – the velocities of all particles in the system

    State `getState`(int *copy*, int *types*, bool *enforcePeriodicBox* = false, int *groups* = 0xFFFFFFFF)¶
    :   Get a `State` object recording the current state information about one copy of the system.

        |  |  |
        | --- | --- |
        | Parameters: |  |

        - **copy** – the index of the copy for which to retrieve state information
        - **types** – the set of data types which should be stored in the `State` object. This should be a union of DataType values, e.g. (State::Positions | State::Velocities).
        - **enforcePeriodicBox** – if false, the position of each particle will be whatever position is stored by the integrator, regardless of periodic boundary conditions. If true, particle positions will be translated so the center of every molecule lies in the same periodic box.
        - **groups** – a set of bit flags for which force groups to include when computing forces and energies. Group i will be included if (groups&(1<<i)) != 0. The default value includes all groups.

    double `getTotalEnergy`()¶
    :   Get the total energy of the ring polymer. This includes the potential and kinetic energies of all copies, plus the potential energy of the harmonic springs that link copies together.

    void `step`(int *steps*)¶
    :   Advance a simulation through time by taking a series of time steps.

        |  |  |
        | --- | --- |
        | Parameters: |  |

        - **steps** – the number of time steps to take

High performance molecular simulation on GPUs


### Navigation

- Core Objects
- Forces
- Integrators
- Extras

©2015, Stanford University and the Authors.
|
Powered by Sphinx 1.3.1
& Alabaster 0.7.7
|
Page source
